# Activity of Cinnamic Acid Derivatives with 4-Chloro-2-mercaptobenzenesulfonamide Moiety against Clinical HLAR and VRE *Enterococcus* spp.

**DOI:** 10.3390/antibiotics12121691

**Published:** 2023-12-02

**Authors:** Rafał Hałasa, Anita Bułakowska, Jarosław Sławiński, Magdalena Smoktunowicz, Aleksandra Rapacka-Zdończyk, Urszula Mizerska

**Affiliations:** 1 Department of Pharmaceutical Microbiology, Medical University of Gdansk, Al. Gen. J. Hallera 107, 80-416 Gdansk, Poland; magdalena.smoktunowicz@gumed.edu.pl (M.S.); a.rapacka-zdonczyk@gumed.edu.pl (A.R.-Z.); 2 Department of Organic Chemistry, Medical University of Gdansk, Al. Gen. J. Hallera 107, 80-416 Gdansk, Poland; anita.bulakowska@gumed.edu.pl (A.B.); jaroslaw.slawinski@gumed.edu.pl (J.S.); 3 Centre of Molecular and Macromolecular Studies, Department of Polymeric Nano-Materials, Polish Academy of Sciences, ul. Sienkiewicza 112, 90-363 Lodz, Poland; urszula.mizerska@cbmm.lodz.pl

**Keywords:** 2-mercaptobenzenesulfonamide, antibiofilm, checkerboard assay, cinnamic acid derivatives, *Enterococcus* spp., synergism

## Abstract

The rapid increase in strains that are resistant to antibiotics requires new active compounds to be found whose mechanism of action on bacteria is different to those that are currently known. Of particular interest are compounds that occur in plants as secondary metabolites. The focus of this study concerns the examination of the effects of synthetic cinnamic acid derivatives, with 4-chloro-2-mercaptobenzenesulfonamide moiety on *Enterococcus* spp. with HLAR (high-level aminoglycoside resistance) and VRE (vancomycin-resistant *Enterococcus*) mechanisms. The minimum inhibitory concentration (MIC) values of the tested compounds were determined using the serial dilution method for *Enterococcus* spp. groups, and the most active compounds were as follows: **16d**, **17c**, **16a**, **16c** and **16f** (2–4 µg/mL). These compounds, at a concentration of 4 × MIC, inhibited the biofilm formation of HLAR strains (70 to 94%). At concentrations of 2 × MIC and 4 × MIC, they also inhibited the growth of VRE strains (42 to 96%). The best effect produced on the formed biofilm was demonstrated by compound **16f** (from 62% MIC concentration to 89% 4 × MIC concentration) on the tested HLAR strains. In vitro studies, using the peripheral blood of domestic sheep, demonstrated the stable bacteriostatic activity of the tested compounds against *Enterococcus* spp. The compounds **16a**, **16c**, **16d**, **16f** and **17c** showed synergism and additivity with ampicillin, streptomycin, gentamicin and vancomycin against resistant strains of *Enterococcus* spp. The tested compounds, when combined, reduce the MIC for antibiotics by 800 to 10,000 times for HLAR strains and by 8 to 10,000 times for VRE strains. The MIC of the tested compounds, in combination with antibiotics, is reduced 2–16-fold for HLAR strains and 2–32-fold for VRE strains. These studies demonstrate the potential for the therapeutic use of synthetic, cinnamic acid derivatives, with 4-chloro-2-mercaptobenzenesulfonamide moiety, to work against clinical strains of *Enterococcus* spp.

## 1. Introduction

The genus *Enterococcus* comprises Gram-positive, catalase negative cocci, usually facultative anaerobic bacteria, that grow in 6.5% NaCl, 40% bile salts, 0.1% methylene blue milk, and at pH 9.6. They also grow at 10 and 45 °C and can survive for 30 min at 60 °C [1]. Enterococci belong to the phylum *Firmicutes* of the *Enterococcaceae* family, which includes many different species, and they are a natural component of the human microbiota. They colonize the lower gastrointestinal tract, oral cavity, and genitals [2]. *E. faecalis* and *E. faecium* are the main enterococci found in the human intestine. *E. cecorum* and *E. durans* are also frequently isolated, whereas *E. casseliflavus*, *E. hirae*, *E. gallinarum*, and *E. avium* are occasionally detected [3]. 

Enterococci can cause a wide range of clinical diseases. One of the factors causing the high infectivity of enterococci is biofilm formation ability. This property makes the treatment of the infection difficult [4]. *Enterococcus* spp. cause infections of the urinary tract, cardiovascular system, abdominal cavity, pelvis, central nervous system, and postoperative wounds. Asymptomatic bacteriuria is the most common clinical condition, but many of these cases are caused by colonization rather than infection. Other frequent causes of infection are bacteremia without endocarditis, which is followed by endocarditis [5]. *Enterococcus* spp. are frequently cited as one of the three most likely etiologies of both uncomplicated and complicated urinary tract infections (UTIs), especially healthcare-associated UTIs. Of these, the bacteria that most commonly cause these infections are *E. faecalis*, although *E. faecium* predominates among vancomycin-resistant isolates. It is usually associated with indwelling urinary catheters and instruments. The severity of the disease can range from uncomplicated cystitis to complicated cystitis, or it may cause pyelonephritis, a perinephric abscess, or prostatitis. All these cases result in the introduction of antibiotics to work against the enterococci [6].

Additionally, enterococci have the ability to obtain genetic material from other bacteria and to quickly adapt to the environment [3]. It is well established that enterococci are resistant to many antibiotics, including the following: β-lactams, aminoglycosides, trimethoprim/sulfamethoxazole, clindamycin, and some species of glycopeptides [7]. This requires combinations of glycopeptides or β-lactams with aminoglycosides to be used [5]. The application of antibiotics in hospitals, and for the treatment of animals, has led to the existence of resistant strains. Currently, drug-resistant enterococci that are isolated in hospitals include the following: high level of resistance to ampicillin; high-level aminoglycoside resistance (HLAR); resistance to glycopeptides (VRE; vancomycin and teicoplanin) and oxazolidinone resistant (LRE—linezolid-resistant *Enterococcus*) [3].

The World Health Organization (WHO) reports that antibiotics are becoming increasingly ineffective as drug resistance spreads around the world, thus causing difficult or ‘impossible to control’ infections. In its reports, the WHO lists microorganisms of particular concern, which require the discovery of new active compounds to work against them; among them are *E. faecium* VRE strains [8,9]. There is a constant need to search for new compounds with antibacterial properties. 

In the light of these issues, research on new drugs to work against resistant bacteria strains is ongoing. An increasing number of studies are being conducted on combination therapies which could potentially reduce the MIC values of administered antibacterial drugs, improve pharmacokinetic/pharmacodynamic (PK/PD) parameters (e.g., bind a hydrophilic drug to a lipophilic drug), and increase activity against bacteria present in biofilm [10]. Examples of such combinations are as follows: omadacycline and oritavancin, or fosfomycin with either daptomycin or rifampicin [11,12].

Secondary plant metabolites (phytochemicals, including cinnamic acid) are a good source of active compounds, including those with antibacterial properties [13]. The broad biological activity of cinnamic acid, and its synthetic derivatives, has been demonstrated by several research teams [9,13,14,15,16,17,18]. Cinnamic acid can occur in nature in both *cis* and *trans* forms with the *trans* form being more stable than the *cis* form. Its antimicrobial activity is low against Gram-positive and Gram-negative bacteria [10]; however, synthetic derivatives (cinnamic acids, esters, amides) have a stronger antimicrobial effect on bacteria; moreover, cinnamic aldehydes inhibit the development of fungi. Cinnamic acid derivatives with phenolic groups have been classified as antioxidants. The chemical structure of cinnamic acid, which contains both a benzene ring and a carboxylic group, enables the modification and procurement of synthetic derivatives. Synthetic compounds that combine two pharmaceutical entities in one molecule may be a successful strategy since these molecules could be more effective than their individual components. Research has been conducted on the conjugation of several hydroxy- and phenyl-substituted derivatives of *trans*-cinnamic acids with an antimicrobial pharmacophore like carvacrol on ESKAPE pathogens [10]. *Trans*-cinnamaldehyde is a phenylpropanoid, which practically occurs in cinnamon essential oil, and it is Generally Recognized as Safe (GRAS) by the US Food and Drug Administration (US FDA) [19].

Sulfonamides and sulfamates are mainly known as synthetic compounds, but several were also discovered as secondary metabolites produced by actinomycetes, e.g., nucleocidin and dealanylascamycin. Sulfonamides and sulfamates constitute a diverse group of highly pharmacologically active compounds, and many clinically used drugs contain the signature sulfamoyl structural motif. Sulfonamides are widely known as synthetic “sulfa drugs”, and these were the first chemotherapeutically used antibacterial compounds. They are applied to other diseases such as Alzheimer’s disease and other central nervous system (CNS) disorders, diabetes, psychosis, and various cancers and tumors [20].

In this work, we examined the activity of synthetic derivatives of cinnamic acid with 4-chloro-2-mercaptobenzenesulfonamide moiety [17] against clinical strains of *Enterococcus* spp. The measure of the activity of compounds is their limit concentration value; for this purpose, we determined minimum inhibitory concentrations. From a clinical point of view, it is important to know: at what concentrations the tested compounds inhibit bacterial biofilm formation, whether they are able to penetrate the already formed biofilm and affect the bacteria or whether they are able to inhibit the further development of the existing biofilm. We examined the activity of new derivatives on enterococcal biofilm. Testing the bioavailability and pharmacodynamics of compounds in body fluids in vitro is one of the important elements showing the stable structure and activity of compounds as well as the influence of the elements of these fluids on the compounds and the impact of the compounds on the components of the higher organism. Therefore, we determined In vitro the stability and influence of blood elements on the antibacterial activity of the tested compounds. Combination therapy of two or three compounds with different targets in a bacterial cell is a common element of antibiotic therapy for infections with multi-resistant strains, including VRE HLAR strains. We investigated the interactions that may occur between the tested compounds and the antibiotics used in the antibiotic therapy of enterococcal infections.

## 2. Results 

### 2.1. Synthesis and Chemical Characterization of Cinnamic Acid Derivatives with 4-Chloro-2-mercaptobenzenesulfonamide Moiety

**Figure 1:**
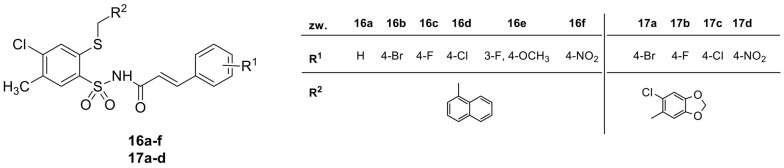
The stuctures of *N*-{[4-chloro-5-methyl-2-(naphthalen-1-ylmethylthio)phenyl]sulfonyl}cinnamamide derivatives (**16a**–**16f**) and *N*-{[4-chloro-2-(6-chlorobenzo[*d*][1,3]dioxol-5-yl)-methylthio-5-methylphenyl]sulfonyl}cinnamamide derivatives (**17a**–**17d**) [18].

The synthesis and chemical characterization of cinnamic acid derivatives with 4-chloro-2-mercaptobenzenesulfonamide moiety was described by Bułakowska et al. [18]. The structures of the tested derivatives are presented in Figure 1.

### 2.2. Antibacterial Activity of Cinnamic Acid Derivatives

In this work, we decided to test the activity of cinnamic acid derivatives against *Enterococcus* characterized by VRE. We preceded the study activity by determining the resistance pattern of all our strains. The studies were performed using the disc diffusion method. The results are presented in Table 1.

It has been observed that all the tested strains were sensitive to linezolid (inhibition zone ≥ 20 mm), and 14 out of 17 bacterial strains were susceptible to teicoplanin (inhibition zone ≥ 16 mm). In turn, 100% HLAR strains were sensitive to vancomycin (inhibition zone ≥ 12 mm). Only one strain showed sensitivity to tetracycline (inhibition zone ≥ 20 mm). However, 100% of the strains tested were resistant to streptomycin (zone of inhibition ≤ 14 mm) and gentamicin (zone of inhibition ≤ 8 mm). Susceptibility to ampicillin (susceptible strain zone ≥ 10 mm; resistant strain ≤ 8 mm), imipenem (susceptible strain zone ≥ 21; resistant strain ≤ 20 mm) and co-trimoxazole (susceptible strain zone ≥ 23 mm) was variable.

The MIC values of the cinnamic acid derivatives with 4-chloro-2-mercaptobenzenesulfonamide moiety against the *Enterococcus* spp. VRE group are shown in Table 2.

The most active compounds turned out to be **16d** and **17c** with MICs within the range of 2 ± 0.05–4 ± 0.38 µg/mL. Compounds **16a** and **16c** showed an MIC of 4 µg/mL. The least active compounds were **17b** and **17d** (MIC > 125 µg/mL). The most resistant strain turned out to be *Enterococcus* sp. 966351, and the most sensitive was *Enterococcus* sp. 885041. The majority of the compounds showed bacterial inhibitory activity, while bactericidal concentrations were equal to or greater than 125 µg/mL. The compounds **16d** and **17c** were the most active against *Enterococcus* spp. VRE.

### 2.3. Effect of Derivatives on the Biofilm Produced by Enterococcus spp. 

**Figure 2:**
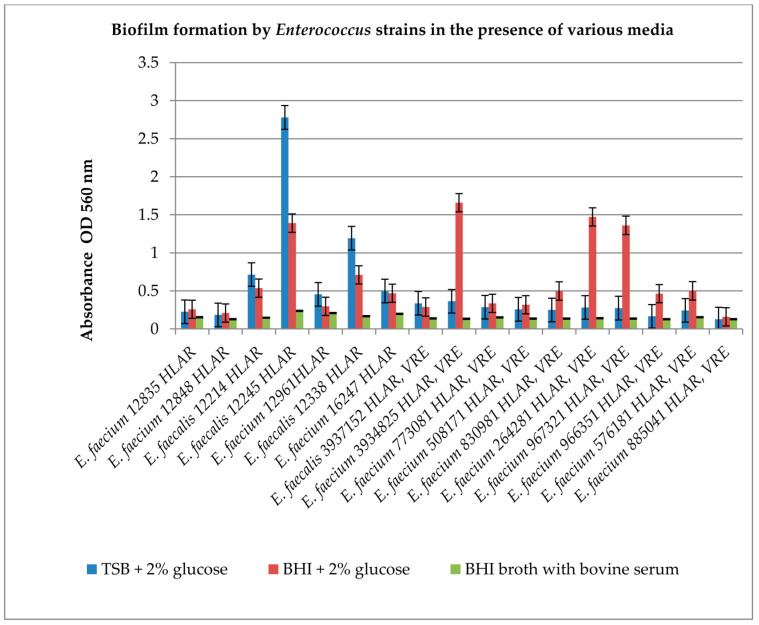
Biofilm formation by *Enterococcus* spp. strains in the presence of various media. The results are presented as mean values ± standard deviation (±SD) from three independent experiments. Error bars represent standard deviation. HLAR (high-level aminoglycoside resistance), VRE (vancomycin-resistant *Enterococcus*) TSB (tryptic soy broth) medium, BHI (brain–heart infusion broth) medium supplemented with 2% glucose and BHI supplemented 5% bovine serum. *p* < 0.05 was considered statistically significant.

The next step of our study was an evaluation of whether the tested cinnamic acid derivatives act on the biofilm produced by *Enterococcus* spp. It is known from the literature data that *Enterococcus* spp. are capable of producing a biofilm, but this is not a permanent feature of the species. Therefore, we decided to verify which of the tested strains exhibit this ability. Based on the literature reports, we chose to test biofilm formation by *Enterococcus* spp., using the following broths: tryptic soy broth medium (TSB), brain–heart infusion broth (BHI) supplemented with 2% glucose, and BHI supplemented with 5% bovine serum. The results of the effect of substrates on biofilm formation are shown in Figure 2.

The results presented in Figure 2 show that the ability to produce biofilm is diversified in the pool of *Enterococcus* spp. strains used for testing in the current study. The strains used include strong biofilm producers, e.g., *E. faecalis* 12245 HLAR and *E. faecalis* 3937152 HLAR and VRE; and those that form a weak biofilm regardless of the substrate used, e.g., *E. faecium* 12835 HLAR and *E. faecium* 12848 HLAR. To the best of our knowledge, it is the first study that differentiates *Enterococcus* according to its ability to form biofilm in different media. The bacteria that generate the strongest biofilm in TSB supplemented with 2% glucose medium are *Enterococcus* spp. HLAR: *E. faecalis* 12214, *E. faecalis* 12245, and *E. faecalis* 12338 (*p* < 0.05). However, in the BHI supplemented with 2% glucose medium, the strongest biofilm is formed by *Enterococcus* spp. VRE: *E. faecium* 3934825, *E. faecium* 967321, and *E. faecium* 264281 (*p* < 0.05). Surprisingly, all the tested strains produced biofilm at a very low level in the presence of bovine serum in the BHI medium (*p* < 0.05). For further research, which concerned the activity of the tested compounds against biofilm, we chose both media and the strains producing the strongest biofilm. The most active derivatives were used in concentrations of 0.5 MIC, MIC, 2 × MIC and 4 × MIC.

**Figure 3:**
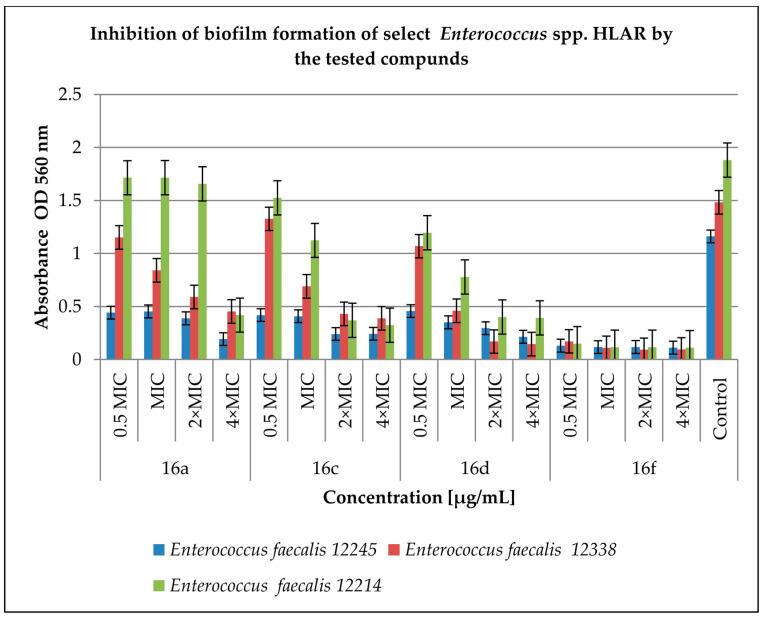
Inhibition of biofilm formation of the selected *Enterococcus* spp. HLAR strains by the tested compounds. A control group was bacterial sample without compounds. Half of the minimum inhibitory concentration—0.5 MIC, double the minimum inhibitory concentration—2 × MIC, quadruple the minimum inhibitory concentration—4 × MIC. The results are presented as mean values ± standard deviation (±SD) from three independent experiments. Error bars represent standard deviation. HLAR (high-level aminoglycoside resistance), VRE (vancomycin-resistant *Enterococcus*). *p* < 0.05 was considered as statistically significant.

Figure 3 shows the effect of the tested derivatives on biofilm formation by *Enterococcus* spp. HLAR strains. The most active compound was **16f**. In the tested concentration range, it significantly inhibited biofilm formation by the *Enterococcus* spp. HLAR strains from 89 to 94% (*p* < 0.05) compared to the control. For the remaining compounds tested, a concentration of 4 × MIC was the most active, as this inhibited biofilm formation from 69% (compound **16a** against *E. faecalis* 12338; *p* < 0.05) to 90% (compound **16d** against *E. faecalis* 12338; *p* < 0.05). The concentrations of 0.5 MIC and MIC turned out to be the least active in inhibiting biofilm formation with the effectiveness of significant effects ranging from 8% (compound **16a** for *E. faecalis* 12214; *p* < 0.05) to 60% (compound **16a** for *E. faecalis* 12245; *p* < 0.05).

**Figure 4:**
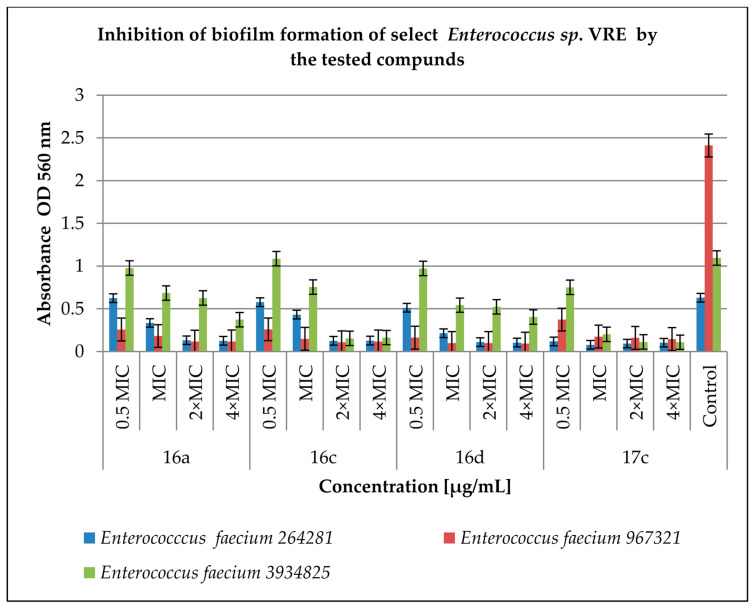
Inhibition of biofilm formation by selected *Enterococcus* spp. VRE by the tested compounds. A control group was bacterial sample without compounds. Half of the minimum inhibitory concentration—0.5 MIC, double the minimum inhibitory concentration—2 × MIC, quadruple the minimum inhibitory concentration—4 × MIC. The results are presented as mean values ± standard deviation (±SD) from three independent experiments. Error bars represent standard deviation. HLAR (high-level aminoglycoside resistance), VRE (vancomycin-resistant *Enterococcus*). *p* < 0.05 was considered as statistically significant.

Figure 4 shows the effect of the tested derivatives on biofilm formation by *Enterococcus* spp. VRE strains. The anti-biofilm activity of the tested compounds varied and depended on the concentration. The inhibition levels of compounds at 0.5 MIC and MIC ranged from 0.78% (compound **16c** at 0.5 MIC against *E. faecium* 3934825; *p* < 0.05) to 95% (compound **16d** at MIC against *E. faecium* 967321; *p* < 0.05). The concentrations of 2 × MIC and 4 × MIC were found to be more effective and inhibited biofilm formation from 42% (compound **16a** at 2 × MIC against *E. faecium* 3934825, *p* < 0.05) to 96% (compound **16d** at 4 × MIC against *E. faecium* 967321; *p* < 0.05) in comparison to the control samples. Comparing the results presented in Figure 2 and Figure 3, it can be concluded that the tested derivatives inhibit the formation of biofilm by the VRE strains more efficiently than by the HLAR strains.

**Figure 5:**
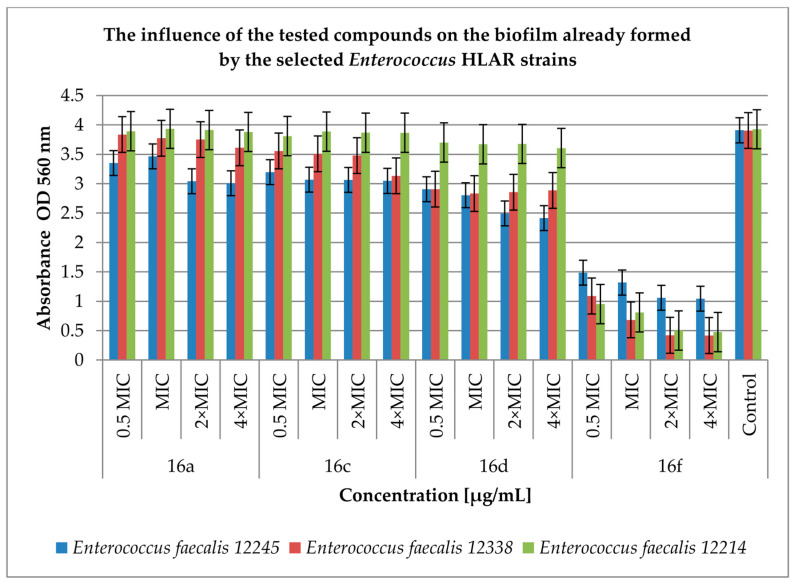
The effect of the tested compounds on the biofilm already formed by selected *Enterococcus* spp. HLAR strains. A control group was bacterial sample without compounds. Half of the minimum inhibitory concentration—0.5 MIC, double the minimum inhibitory concentration—2 × MIC, quadruple the minimum inhibitory concentration—4 × MIC. The results are presented as mean values ± standard deviation (±SD) from three independent experiments. Error bars represent standard deviation. HLAR (high-level aminoglycoside resistance), VRE (vancomycin-resistant *Enterococcus*. *p* < 0.05 was considered as statistically significant.

**Figure 6:**
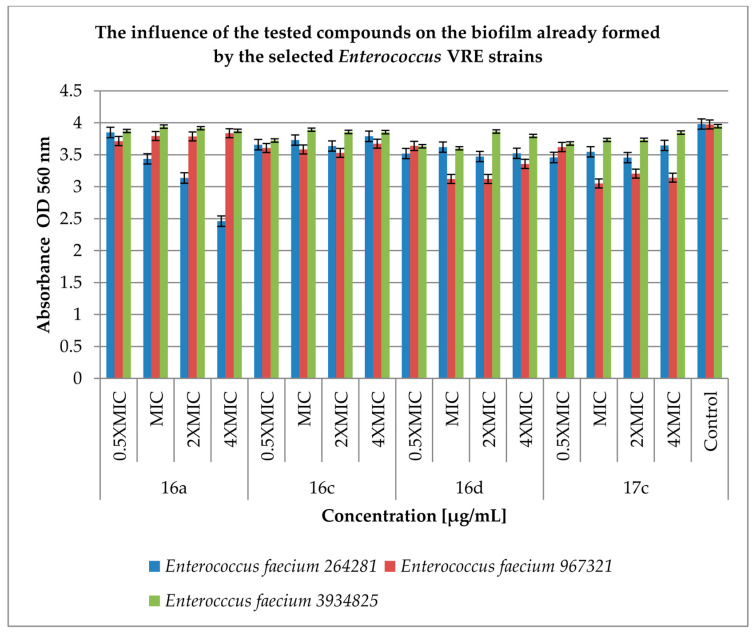
The effect of the tested compounds on the biofilm already formed by selected *Enterococcus* spp. VRE strains. A control group was bacterial sample without compounds. Half of the minimum inhibitory concentration—0.5 MIC, double the minimum inhibitory concentration—2 × MIC, quadruple the minimum inhibitory concentration—4 × MIC. The results are presented as mean values ± standard deviation (±SD) from three independent experiments. Error bars represent standard deviation HLAR (high-level aminoglycoside resistance), VRE (vancomycin-resistant *Enterococcus. p* < 0.05 was considered as statistically significant.

The next step of our work was to test the antimicrobial effect of our derivatives on already formed enterococcal biofilm. The results are presented in Figure 5 and Figure 6.

Figure 5 shows the effect of the tested compounds on biofilm already formed by *Enterococcus* spp. HLAR strains. Compounds **16a**, **16c** and **16d** show a low effect on the already formed biofilm and the degree of reduction in the tested strains in biofilm ranged from 0.8% (compound **16a** at a concentration of 0.5 MIC against *E. faecalis* 12214; *p* < 0.05) to 38% (compound **16d** at a concentration of 4 × MIC against *E. faecalis* 12245; *p* < 0.05) when compared to the control. In contrast, compound **16f** turned out to most actively reduce the number of bacteria in the biofilm from 62% (0.5 MIC concentration against *E. faecalis* 12245; *p* < 0.05) to 89% (4 × MIC concentration against *E. faecalis* 12338; *p* < 0.05) compared to the control samples.

Nevertheless, the tested compounds had no significant effect on the inhibition of further biofilm formation by the *Enterococcus* spp. VRE strains (Figure 6). Inhibitory activities ranged from 0.16% (compound **16a** at MIC concentration against *E. faecium* 3934825; *p* < 0.05) to 23% (compound **17c** at MIC concentration against *E. faecium* 967321; *p* < 0.05) compared to the control.

The most active compounds were **16f** and **17c**. However, the activity of the tested compounds on the already formed biofilm was low in both groups of bacteria with the exception of compound **16f** acting on *Enterococcus* spp. HLAR, which inhibits the formation of the biofilm to about 90% when compared to the control.

### 2.4. Blood Bacteriostatic Activity Tests

The compounds we tested exhibited a bacteriostatic effect on *Enterococcus* spp. in the culture medium. We wanted to answer the question of whether this property is retained in the presence of blood components, as it was in the case of *Staphylococcus* spp. [18]. For this purpose, we selected two species of bacteria: *E. faecium* 264281 (Table 3) and *E. faecalis* 12245 (Table 4).

The initial number of bacteria at time t_0_ was (2.00 ± 0.69) × 10^8^ CFU/mL for *E. faecalis* 12245 and (7.30 ± 0.67) × 10^8^ for *E. faecium* 264281. Bacteria in appropriate numbers were added to blood samples containing the tested compounds in the concentration range of 0.5–16 µg/mL and then incubated for 24 h at 37 °C. Controls consisted of samples of bacteria incubated in pure blood, without supplements, the number of which after 24 h incubation was (1.44 ± 0.47) × 10^8^ CFU/mL for *E. faecalis* 12245 and (1.30 ± 0.48) × 10^8^ CFU/mL for *E. faecium* 264281. There is a relationship here: the higher the concentration of the active compound in the sample, the lower the number of live bacteria. In the samples with compounds **16c** and **16d** (16 µg/mL), there is a significant decrease in the number of viable bacteria: **16c**—(7.00 ± 0.71) × 10^6^ CFU/mL, **16d**—(1.20 ± 0.55) × 10^6^ CFU/mL (*p* < 0.05) compared to the control. 

### 2.5. Study of Interactions of Cinnamic Acid Derivatives with Antibiotics on Resistant Strains of Enterococcus spp.

The most interesting interactions between antibacterial compounds relate to synergy. The synergy of compounds is based on increasing effectiveness, reducing toxicity, reducing undesirable side effects, increasing bioavailability, lowering the required dose and limiting the emergence of resistance to the tested preparations. New therapeutic combinations that include a connection with natural products have become a research priority. 

To check the relationship between our derivatives and antibiotics, we conducted further studies using the common checkerboard assay. Antibiotics such as ampicillin, gentamicin, streptomycin and vancomycin as well as *Enterococcus* spp. selected from both tested groups were used.

In the first stage, we checked the activity of selected antibiotics and determined the limit concentrations (MIC). In the next stage, we conducted research assessing the relationship between the tested derivatives and selected antibiotics, where we used MIC and lower concentrations of the tested compounds. The antibiotics taken into consideration in the study are important preparations in the treatment of infections caused by *Enterococcus* spp. The results are presented in Table 5 and Table 6.

The cinnamic acid derivatives used in the research show a synergistic effect with antibiotics against selected HLAR strains. The exception is compound **16d**, which presents additive properties with ampicillin and gentamicin. Compounds **16a**, **16c** and **16f** are synergistic compounds with the tested antibiotics. These compounds may become potential preparations for clinical use in combination with currently used antibiotics against HLAR strains. However, the results obtained for selected VRE strains are not as homogeneous as for HLAR strains. Most of the tested compounds in a mixture with antibiotics show synergism. However, some compounds (**16a**, **16c**, **16d** and **17c**), when combined with antibiotics (gentamicin, streptomycin and vancomycin), give additive effects. Compound **16a** exhibits synergistic properties with all the tested antibiotics against selected VRE strains. In turn, all the compounds show synergy with ampicillin. The remaining tested compounds (**16c**, **16d** and **17c**) show differentiated interactions with antibiotics: synergism or additivity.

## 3. Discussion 

In our previous study, we showed that cinnamic acid derivatives with 4-chloro-2-mercaptobenzenesulfonamide moiety were active against reference and clinical strains of *Enterococcus* spp. The tested enterococci strains were mostly HLAR and included both *E. faecalis* and *E. faecium* strains. Only two of the tested isolates were VRE strains. In this work, we decided to test the activity of cinnamic acid derivatives against *Enterococcus* characterized by VRE.

The results of our previous research conducted by Bułakowska et al. [18] showed that the most active derivatives against *Enterococcus* spp. HLAR were **16a**, **16c**, **16d** and **16f**. Compounds **16d** and **17c** were the most active against *Enterococcus* spp. VRE. Both derivatives contain a chlorine atom as the substituent R^1^ and naphthyl or piperonyl as the R^2^ substituent. Moreover, derivatives **16a** and **16c** turned out to be very active against *Enterococcus* spp. VRE as well as for HLAR strains. Derivative **16f** was less active against VRE than against HLAR strains.

Based on the obtained results, it can be concluded that the activity of the tested derivatives against *Enterococcus* spp. HLAR and VRE was mainly due to the presence of the substituent R^2^—naphthyl, and the substituent R^1^ = H, F, NO_2_ was secondary. The high activity of compounds **16d** and **17c** against *Enterococcus* spp. of the VRE group could mean that the substituent R^1^ = Cl determines the main activity of the compound with the substituent R^2^ only in the second place. When comparing the MIC values, obtained by serial dilution, to the standard (co-trimoxazole), the tested compounds turned out to be significantly more active against the tested *Enterococcus* strains. This is most likely related to the structure of the compounds—the combination of sulfonamide with cinnamic acid in one molecule and a different mechanism of action on microorganisms than the composition of co-trimoxazole.

Mingoia et al. [10] investigated the antibacterial properties of several hydroxy- and phenyl-substituted *trans*-cinnamic acid derivatives conjugated with an antimicrobial pharmacophore such as carvacrol in order to select the best candidate for the treatment of cutaneous infections caused by pathogens. Among the list of derivatives, only DM2 and DM8 turned out to be active against the tested bacterial strains. Compound DM2 was more active against *E. faecium* (MIC_50_ 32 mg/L) than *E. faecalis* (MIC_50_ 256 mg/L). The authors [9] suggest that the presence of a hydroxyl group in the *ortho* position in the phenyl nucleus is important for the effect on the cell membrane of enterococci. Therefore, the DM8 compound turned out to be the most active against *E. faecium* (MIC_50_ = 32 mg/L). It needs to be highlighted that the authors themselves, in Table 2, presented the range of the antibacterial activity of selected derivatives, including MD8, which for *E. faecalis* was in the range of 32–512 mg/mL and for *E. faecium* was in the range of 16–512 mg/mL. Hence, the authors’ suggestion as to the mechanism is not that clear.

Guzman et al. [15] reported that cinnamic acid was active against *E. faecalis* and its minimum inhibitor concentration was 6.75 mM. In turn, the MICs of ferulic acid and its 4-*O*-acetyl derivative for *E. faecalis* ATCC 29212 were determined as 659 μM and 540 μM, respectively. Among natural and synthetic cinnamic esters, activity was demonstrated by 5-*O*-caffeoylquinic acid (MIC 181 μM) against *E. faecalis* OGRF1, rosmarinic acid (MIC 833 μM) and methyl rosmarinate (MIC 801 μM) against *E. faecalis* C159-6, and finally caffeic acid phenethyl ester (MIC 400 μM) against *E. faecalis* ATCC 29212. 4-*tert*-butylphenyl ferulate and 4-isopropylphenyl ferulate were active against *E. faecalis* ATCC 29212 at the concentrations of 391 μM and 51 μM, respectively. In turn, the MIC of 4-chloro-3-methylphenyl ferulate was 50 μM. Among cinnamic aldehydes, alcohols and their derivatives, the most active against *E. faecalis* turned out to be cinnamaldehyde at a concentration of 1.89 mM. 

Many research groups are engaged in the construction of hybrid compounds using cinnamic acid. Cephem hybrid with cinnamic aldehyde gave results of activity against *E. faecalis* A20688 as 1.6 μM, 1.5 μM, 1.4 mM and 364 nM. Oxazolidinone hybrids are active against *E. faecium* ATTC 700221 (VRE; MIC 388 nM) and *E. faecalis* ATCC 29212 (MIC 194 nM) [15].

Gómez et al. [21] examined the antimicrobial properties of natural essential oil components, including cinnamaldehyde, against three species of bacteria: *Enterococcus* spp., *Staphylococcus* spp., and *Pseudomonas* spp. (10 clinical strains of each). Cinnamaldehyde (CIN) presented the highest activity for all tested bacterial strains—the MICs within the range of 10–50 mg/mL. The research was conducted in Mueller Hinton (MH) broth or cation-adjusted Mueller Hinton II broth (MH II).

Teethaisong et al. [22] investigated the effect of *Stephania suberosa* extract (SSE) against *E. faecium* strains and obtained MIC values of 0.5 mg/mL.

Biofilm consists of organized spaces, a multi-layer cluster of cells of one or several species of microorganisms, formed on biotic and abiotic surfaces. Microorganisms concentrated in biofilm are characterized by diverse metabolic activity. There are also antibiotic-resistant bacteria that “protect” the susceptible bacteria. Biofilm-forming bacteria are surrounded by a special extracellular polymeric substance (EPS) matrix. It is already known that Enterococcal surface protein (ESP) significantly enhanced *E. faecalis* biofilm formation in a glucose-dependent manner [22]. Biofilm is the cause of many life-threatening infections of humans and animals. Due to its complex structure, it is very difficult to remove; EPS is difficult for disinfectants and antibiotics to penetrate.

The compounds **16a**, **16c**, **16d**, **16f** and **17c** were tested at concentrations of 0.5 MIC, 2 × MIC and 4 × MIC and inhibited the formation of biofilm by both groups of enterococci, but the inhibition of the VRE strains was more efficient than that of the HLAR strains. However, the activity of the tested compounds against the already formed biofilm was low in both groups of bacteria; only compound **16f** was active against *Enterococcus* spp. HLAR and inhibited the formation of the biofilm to about 90% in comparison to the control. In our studies, we determined the amount of biofilm with crystal violet, as was previously described in [18].

Ali et al. [19], using BHI medium, examined the effect of *trans*-cinnamaldehyde (0.5%, 0.75% and 1%) on the biofilm formed by *E. faecalis* (clinical isolate) for 72 h. The authors treated the biofilm with the compounds for 5 and 15 min or 24 h and demonstrated, using 2,3-bis-(2-methoxy-4-nitro-5-sulfophenyl)-2*H*-tetrazolium-5-carboxanilide (XTT), that a 15-min action of cinnamaldehyde kills bacterial cells. Derivatives studied in our work had a bacteriostatic effect on planktonic bacterial cells in the tested concentration range. Due to the higher resistance of biofilm cells to chemicals than free-living cells, we chose crystal violet for biofilm determination in our studies. Crystal violet shows the size (biomass) of the biofilm presence or its absence rather than the presence of living cells, as was described in Ali et al. [19].

Akshaya et al. [23] examined the effect of cinnamaldehyde in the concentration range of 62.5–1000 μM on the biofilm formed by the *E. faecalis* strain (MTCC 2729) using BHI medium with 2% glucose for 72 h. The researchers used crystal violet and XTT [2,3-bis-(2-methoxy-4-nitro-5-sulfophenyl)-2*H*-tetrazolium-5-carboxanilide] as markers. Cinnamaldehyde prevented the growth of *E. faecalis* biofilm from a concentration of 62.5 μM, according to the crystal violet-staining technique. It was found that cinnamaldehyde at a concentration of 1000 µM inhibited the production of *E. faecalis* biofilm by 85%.

Akshaya et al. [23] studied the effect of cinnamaldehyde on the expansion and gelatinase activity of *E. faecalis* strains, which is important in the biofilm formation process. Gelatinase activity has been reported also to be involved in the initial steps of the generation of biofilm. In our studies, we detected phenotypically the presence of gelatinase produced by the tested strains. Unfortunately, we do not notice a correlation between the ability to form a biofilm and the presence of enzymes. The gelatinase activity was demonstrated by the following strains: *E. faecalis* 12245, 12214, 12338, 3937152 and *E. faecium* 264281, 508171, 16247; these strains formed a strong or a weak biofilm, as shown in Figure 1.

Hashem et al. [24] showed, using *gelE(*–) mutants, that the lack of the enzyme did not result in the lack of biofilm formation, which is consistent with our results.

The literature states [23] that the biofilm-forming activity of *E. faecalis* in the presence of glucose varies. Based on our results (Figure 2) (e.g., in the case of *E. faecalis* 12214, 12245, 12338, 12961), the presence of 2% glucose in two different media did not result in biofilm formation at the same level. There must be other factors that influence biofilm formation, depending on the medium used.

Enterococci possess in their genome the *fsr* locus that shares significant homology with the *agr* locus of staphylococci, which acts as an accessory global regulator of virulence factors and metabolism, including biofilm formation. Pillai et al. [25] showed that biofilm formation by *E. faecalis* OG1RF was greater in TSB supplemented with 1% glucose, but the same effect was not evident in *fsr*, *gelE* or *sprE* mutants. This could suggest that a glucose-dependent transcriptional regulator controlled *fsr*, either directly or indirectly, and that *fsr* exerts catabolite control over biofilm formation through GelE and SprE downstream proteases.

Kim et al. [26] examined the effect of tryptone–yeast extract broth with 0% glucose + 0% sucrose, 0.5% glucose, 1% glucose, 0.5% sucrose or 1% sucrose on *Enterococcus faecalis* planktonic and biofilm In vitro. They showed that the virulence-associated gene expression was the highest in broth with 1% sucrose (biofilm growth conditions) and in broth with 1% glucose (planktonic growth conditions). A higher bacteria and exopolysaccharide (EPS) bio-volume in sucrose was observed than in 0% glucose + 0% sucrose or glucose.

Interestingly, many researchers study the impact of chemical compounds on the already formed biofilm but do not check the preventive effect. Certainly, destroying the formed biofilm is a very important issue in the search for compounds that act on bacteria, but the administration of preventive preparations or coating materials used for catheterization, drainage, etc., may also be an important element in the fight against infections with resistant strains.

Caballero Gómez et al. [21], using TSB medium, examined the effect of natural essential oil components (including cinnamaldehyde) at MIC and 0.5 MIC concentrations, and their combination with EDTA (12.5–25 mM in water) and HLE (disinfectant solution—composed of 3–6% H_2_O_2_; 2.2–4.4% lactic acid) on the biofilm formed by selected strains. Crystal violet was used to determine the presence of biofilm. Cinnamaldehyde inhibited the formation of *Enterococcus* sp. strain M28M12 biofilm by 80%; for the other two strains, the effect was not so spectacular. However, a synergistic effect between cinnamaldehyde with HLE and EDTA was visible. CA + HLE caused 2–6 log_10_ reductions in CFU (colony-forming units) of enterococci and CA + EDTA 2–3 log_10_ reductions in CFU of enterococci in biofilm.

The presence of whole peripheral blood (Method 4.5) with its antibacterial mechanisms and the presence of phagocytic cells may have resulted in a slight decrease in the number of bacteria in the control samples. It seems that a similar mechanism occurred in the case of the lowest concentrations (0.5 µg/mL) of the tested compounds. This may be due to the fact that *Enterococcus* spp. do not have many virulence factors, like staphylococci, that neutralize the defense mechanisms present in the blood of mammals. In tests with higher concentrations of compounds, the number of viable bacterial cells changes significantly. These changes are a combination of the mechanisms of the immune system present in the blood and the bacteriostatic effect of the concentrations of compounds. The results obtained may be due to the influence of the tested derivatives on delays in the division of bacterial cells, which, when transferred to a solid substrate without the tested agent, have an extended doubling time and did not produce visible colonies even after 48 h of incubation. We suggested that the tested compounds have constant, stable bacteriostatic activity in whole blood, and they do not inhibit the activity of the cells of the immune system. This could be a basis for further research into potential drugs against resistant bacteria.

Doyle et al. [27] cite that *trans*-cinnamaldehyde has properties that limit its use: low solubility in water, sensitivity to light and air, and it is not very stable in the blood where it is converted into cinnamic acid. Present in cosmetics in concentrations above 0.005%, it causes allergies and adverse reactions. The authors emphasize the need to test aldehyde derivatives for toxicity. The derivatives we tested show toxic activity to the peripheral blood of domestic sheep at concentrations of 32 µg/mL, as we demonstrated in the publication by Bułakowska et al. [18]. These compounds present stable activity against MRSA and *Enterococcus* spp. strains (data above) in the tested concentration range, during 24 h of testing. However, it would be necessary to carry out tests on the stability of the antimicrobial activity of compounds in biological systems over an extended period of time and to transform the derivatives we studied into water-soluble forms.

*Enterococcus* spp. are bacteria that are naturally resistant to many antibiotics, and the treatment of infections is mainly based on combining several antibiotics in order to achieve a synergistic effect. This effect is intended to fight the infection caused by multidrug-resistant strains. We wanted to check whether our compounds have any interactions with conventional antibiotics.

The cinnamic acid derivatives used in the research show a synergistic effect with antibiotics against selected HLAR strains. The exception is compound **16d**, which presents additive properties with ampicillin and gentamicin. It should be emphasized here that the tested compounds reduce the MIC for antibiotics by 800 to 10,000 times. However, the MIC of the tested compounds in combination with antibiotics is reduced by 2 to 16 times. Compounds **16a**, **16c** and **16f** are compounds that act synergistically with the tested antibiotics. These compounds may become potential preparations for clinical use in combination with currently used antibiotics against HLAR-resistant strains. However, the results obtained for selected VRE strains are not as homogeneous as for HLAR strains. Most of the tested compounds in a mixture with antibiotics show synergism. However, some compounds (**16a**, **16c**, **16d** and **17c**), when combined with antibiotics (gentamicin, streptomycin and vancomycin), give additive effects. The tested compounds reduce the MIC of antibiotics by 8 to 10,000 times. However, the combination of the tested derivatives with antibiotics causes a decrease in the MIC values of the compound by 2 to 32 times. Compound **16a** exhibits synergistic properties with all the tested antibiotics against selected VRE strains. In turn, all the compounds show synergy with ampicillin. The next stage of further research would be to check the activity of concentrations showing synergism, how they act on bacteria over time, how they affect biofilm and whether their activity is stable in the blood.

Essential oil (from *C. zeylanicum*) exhibits synergistic activity with amikacin, gentamicin, imipenem and meropenem against *Acinetobacter baumannii* and has a positive interaction with colistin [26]. Essential oil obtained from *Cinnamomum burmannii* interacts with gentamicin against *S. epidermidis,* and *Cinnamomum verum* essential oil showed the ability to restore the sensitivity of *Escherichia coli* to piperacillin (the bacterium had the TEM-1 beta-lactamase gene) [26]. *Trans*-cinnamaldehyde from *C. zeylanicum* decreased the MIC of clindamycin for *Clostridium difficile* by 16-fold [26].

Doyle et al. [27] presented a report on the synergism of cinnamaldehyde in combination with ampicillin, bacitracin, clindamycin, erythromycin, novobiocin, penicillin, streptomycin, sulfamethoxazole and tetracycline against *Salmonella* Typhimurium SGI1 (*tet A*), *Escherichia coli* N00-666, *Staphylococcus aureus blaZ*, which was resistant to penicillin, and erythromycin-resistant *Streptococcus pyogenes ermB*.

Cinnamic acid showed synergism with amikacin, ampicillin, ciprofloxacin, erythromycin, or vancomycin against *E. coli* and *S. aureus* and a combination of cinnamic acid with ciprofloxacin against *P. aeruginosa*. Cinnamic acid displays synergistic effects with amikacin, clofazimine, isoniazid and rifampin against *Mycobacterium tuberculosis* and *M. avium* [27].

Teethaisong et al. [22], using the checkerboard method, determined that SSE plus ampicillin and SSE plus vancomycin combinations exhibited synergistic interactions against *E. faecium* isolates.

## 4. Materials and Methods

### 4.1. Materials

Sterile sheep blood was defibrinated (GrasoBiotech, Starogard Gdański, Poland). Co-trimoxazole (composition sulfamethoxazole 400 mg: trimethoprim 80 mg; WZF Polfa, Warszawa, Poland), ampicillin-Na-salt (Serva, Heidelberg, Germany), streptomycin sulfate salt (Pol-Aura, Dywity, Poland), gentamycin (40 mg/mL, solution for injection and infusion; (KRKA d.d., Novo mesto, Slovenia), amikacin disulfate salt (Pol-Aura, Dywity, Poland), norfloxacin (Pol-Aura, Dywity, Poland), levofloxacin (5 mg/mL, solution for infusion, (Pharmathen S.A, Pallini, Greece), vancomycin hydrochloride (Pol-Aura, Dywity, Poland), ciprofloxacin (10 mg/mL, solution for infusion, (KRKA d.d., Novo mesto, Slovenia), vancomycin hydrochloride (Pol-Aura, Dywity, Poland), doxycycline (20 mg/mL, solution for infusion (Polfa Tarchomin S.A., Warszawa, Poland). Brain–heart infusion broth (BHI, Becton Dickinson, Franklin Lakes, NJ, USA) was used for MIC and FICI determination, while BHI and tryptic soy broth (TSB, Becton Dickinson, Franklin Lakes, NJ, USA) medium supplemented with 2% glucose were used to culture the biofilm bacteria. Muller–Hinton agar (MH, Becton Dickinson, Franklin Lakes, NJ, USA) was used alongside the disc diffusion method. Bacterial strains and culture conditions: lists of strains tested in the publication are presented in Table 7a, b. *Enterococcus faecalis* ATCC 51299 and clinical strain *Enterococcus* spp. were cultured in an aerobic atmosphere at 37 °C for 48 h. To determine the bacterial viability, BHI blood agar plates were used.

### 4.2. Determination of Susceptibility to Antibiotics by the Agar Disc Diffusion Method

Disc diffusion methods for antibiotics [28] and antibiotic susceptibility were interpreted according to EUCAST clinical breakpoints (version 11.0) [29]. In our research, Muller–Hinton medium (Beckton-Dicinson, Franklin Lakes, NJ, USA) and antibiotic paper discs (BioMaxima S.A., Lublin, Poland, Country) like ampicillin 2 µg/mL, imipenem 10 µg/mL, norfloxacin 10 µg/mL, vancomycin 5 µg/mL, teicoplanin 30 µg/mL, co-trimoxazole 25 µg/mL, doxycycline 30 µg/mL, linezolid 10 µg/mL, gentamicin 120 µg/mL, and streptomycin 300 µg/mL were used.

### 4.3. Minimum Inhibitory Concentration Determination

The MIC determination for the reference and clinical strains was performed based on the methodology described by Bułakowska et al. [18]. The dry test samples were dissolved in dimethyl sulfoxide (DMSO) and diluted in water, resulting in a final concentration of about 500 µg/mL. Antibiotics were weighed and dissolved in water, resulting in a final concentration of ampicillin (200 mg/mL), streptomycin (200 mg/mL), amikacin (80 mg/mL), norfloxacin (40 mg/mL) and vancomycin (80 mg/mL). The other antibiotics were in concentrations: co-trimoxazole (composition sulfamethoxazole 400 mg: trimethoprim 80 mg), gentamycin (40 mg/mL), levofloxacin (5 mg/mL), ciprofloxacin (10 mg/mL), and doxycycline (20 mg/mL). These solutions were diluted and added to the first well of each microtiter line. Dilution in geometric progression was completed by transferring the mixture/dilution (100 μL) from the first to the twelfth well. An aliquot (100 μL) was discarded from the twelfth well. Then, 100 μL of bacterial suspension was added to each well. The final concentration of the synthetic compound used in the antimicrobial activity assay ranged from 125 to 0.006 µg/mL. The final concentration of the antibiotics was: ampicillin (from 10 to 0.05 mg/mL), streptomycin (from 10 to 0.05 mg/mL), gentamycin (from 2 to 0.001 mg/mL), amikacin (from 4 to 0.002 mg/mL), norfloxacin (from 2 to 0.001 mg/mL), levofloxacin (from 0.25 to 0.000125 mg/mL), ciprofloxacin (from 0.5 to 0.00025), vancomycin (from 4 to 0.002 mg/mL) and doxycycline (from 1 to 0.0005 mg/mL). Tests were incubated in adequate conditions described by Bułakowska et al. [18]. The MIC was considered the lowest concentration at which no visible growth was observed. Diluent concentration had no effect on the activity of the tested compounds. All experiments were carried out three times.

### 4.4. Inhibition of Biofilm (Prior to Biofilm and Post-Biofilm) Formation by the Cinnamic Acid Derivatives

Determination of the biofilm inhibitory concentration (before and after biofilm formation) was carried out according to the method described by Bułakowska et al. [18] with modification. For the determination of biofilm, we used TSB (tryptic soy broth) supplemented with 2% glucose, BHI (brain–heart infusion broth) medium supplemented with 2% glucose, and BHI medium supplemented with 5% bovine serum. We also increased the range of concentrations of the tested compounds of 0.5 × MIC, MIC, 2 × MIC and 4 × MIC. The final volume of the mixture was 200 µL. In the post-biofilm formation, biofilms were formed for 24 h at 37 °C, non-adherent cells were removed, and compounds at the concentrations of 0.5 × MIC, MIC, 2 × MIC and 4 × MIC were added into each well. After 24 h incubation at 37 °C, the contents of each well were discarded and washed 3 times with sterile deionized water in order to remove non-adherent cells. The biofilm was fixed with 2% formaldehyde (0.5 h) and then stained with 0.1% crystal violet solution for 1 h at 37 °C. Next, plates were rinsed with water until clean drops were obtained. The stained biofilm was dissolved by 96% ethanol, and growth of bacteria was quantified by measuring the OD at 560 nm using a microplate reader (Infinite^®^ 200 PRO, Tecan, Männedorf, Switzerland). Positive control were bacteria without compounds. All experiments were carried out in three repetitions.

### 4.5. Blood Bacteriostatic Activity Tests

The assay was performed as previously described by Bułakowska [18] with a modification. Pure sheep blood was used for the research. Two strains of bacteria *E. faecalis* 12245 and *E. faecium* 264281 were used with an inoculum density of approximately 10^8^ CFU/mL at time 0 (t_0_). The final concentration of the compounds were 0.5, 1, 2, 4, 8 and 16 µg/mL, and the final volume of the assay tube was 1 mL. Bacterial survivors were determined on BHI agar plates (CFU/mL) at 24 h after exposure. All experiments were carried out in three repetitions.

### 4.6. Checkerboard Arrays for Planktonic Bacteria (Fractional Inhibitory Concentration Index)

The checkerboard arrays method was described in [22]. The BHI broth was used in the studies. Antibiotics (ampicillin, gentamicin, streptomycin, vancomycin) in the range of 16 to 1 µg/mL and tested compounds in the range of 8 to 0.5 µg/mL were tested. Bacteria were cultured 48 h in aerobic conditions. After incubation, the turbidity of the samples was visually determined and the results were read. The MICs of the antibiotic and the test compound were determined and substituted into the formula to calculate the Fractional Inhibitory Concentration Index (FICI):

FICI = FIC_A_ + FIC_B_ = A/MIC_A_ + B/MIC_B_

MIC_A_ and MIC_B_ are the MICs of drugs A and B alone, respectively. A and B are the concentrations of the drugs in combinations, respectively. FIC (index) values were interpreted as follows [30]: FIC index ≤ 0.5 indicates synergism, FIC index > 0.5 to < 1.0 shows partial synergism, FICI = 1.0 indicates addition, FIC index > 1.0 to 4.0 denotes indifference, and FIC index > 4.0 denotes antagonism [22].

### 4.7. Statistical Analysis

All experiments were performed at least three times. The intergroup differences were estimated by one- or two-way analysis of variance by statistical package Microsoft Excel 2010. The data are presented as mean and standard deviation (SD). A *p*-value was considered as statistically significant when it was less than 0.05.

## 5. Conclusions

In the current study, we continued the studies on cinnamic acid derivatives with 4-chloro-2-mercaptobenzenesulfonamide moiety on Gram-positive cocci—*Enterococcus* spp. We expanded the number of research objects; in addition to clinical strains with HLAR (MIC **16d**, **17c**, **16a** and **16c** values are published by Bułakowska et al. [18]), we added the strains with the VRE mechanism of resistance and determined the active concentrations of the tested compounds against these bacteria. The most active compounds for the group of VRE strains were **16c**, **17c**, **16a** and **16d**. Whereas, for the HLAR group, the most active turned out to be **16a**, **16c**, **16d** and **16f**. Compounds **16d** and **17c** contain two or three chlorine atoms per molecule, and a strong effect may result from HLAR and VRE bacterial cells. Compounds **16f**, **16a**, **16c** and **16d** were the most active and inhibited biofilm formation by the tested *Enterococcus* spp. HLAR strains. The biofilm formation of VRE strains was inhibited by **16a**, **16c**, **16d** and **17a**. Compounds **16a**, **16c** and **16d** showed a low effect on the already formed biofilm of the tested strains; only compound **16f** appeared to be the most effective and inhibited the biofilm. We have shown that the tested derivatives maintain stable activity, inhibiting the growth of bacteria in the peripheral blood of domestic sheep. The interactions between antibiotics and compounds **16a**, **16c** and **16f** presented a synergistic effect with ampicillin, gentamicin, streptomycin and vancomycin against HLAR strains. The compounds **16a**, **16c**, **16d** and **17c** exhibit synergism, partial synergism or additivity against VRE strains. It should be noticed that the tested compounds reduce the MIC for antibiotics by 8 to 10,000 times. The MIC of the tested compounds in combination with antibiotics is reduced by 2 to 32 times. In summary, our results indicate that the tested cinnamic acid derivatives may be promising in the treatment of bacterial infections.

## Figures and Tables

**Table 1:** Phenotypic characterization of resistance of *Enterococcus* strains. Inhibition zone diameters around antibiotic discs (mm).

Full Strain Name, and Specific Resistance Mechanism	Ampicillin	Imipenem	Vancomicin	Teicoplanin	Co-trimoxazole	Norfloxacin	Gentamicin	Streptomycin	Doxycycline	Linezolid
*Enterococcus faecium* 12835 HLAR	6 ± 0.00	6 ± 0.00	15 ± 0.00	20 ± 0.50	6 ± 0.00	6 ± 0.00	6 ± 0.00	6 ± 0.00	6 ± 0.00	22 ± 0.0
*Enterococcus faecium* 12848 HLAR	6 ± 0.00	6 ± 0.00	17 ± 0.50	18 ± 0.50	6 ± 0.00	6 ± 0.00	6 ± 0.00	6 ± 0.00	6 ± 0.00	25 ± 0.00
*Enterococcus faecalis* 12214 HLAR	11 ± 0.50	21 ± 0.00	15 ± 0.00	20 ± 0.50	40 ± 1.00	6 ± 0.00	6 ± 0.00	6 ± 0.00	6 ± 0.00	19 ± 0.00
*Enterococcus faecalis* 12245 HLAR	12 ± 0.50	22 ± 0.00	15 ± 0.00	18 ± 0.50	40 ± 1.00	6 ± 0.00	6 ± 0.00	6 ± 0.00	6 ± 0.00	25 ± 0.00
*Enterococccus faecium* 12961 HLAR	6 ± 0.00	6 ± 0.00	12 ± 0.00	20 ± 0.50	41 ± 1.00	6 ± 0.00	6 ± 0.00	6 ± 0.00	20 ± 0.00	26 ± 0.00
*Enterococcus faecalis* 12338 HLAR	12 ± 0.50	21 ± 0.00	15 ± 0.00	19 ± 0.00	35 ± 1.00	6 ± 0.00	6 ± 0.00	6 ± 0.00	6 ± 0.00	25 ± 0.50
*nterococcus faecium* 16247 HLAR	6 ± 0.00	6 ± 0.00	12 ± 0.00	20 ± 0.50	6 ± 0.00	6 ± 0.00	6 ± 0.00	6 ± 0.00	6 ± 0.00	26 ± 0.00
*Enterococcus faecalis* 3937152 HLAR, VRE	14 ± 0.50	22 ± 0.00	6 ± 0.00	20 ± 0.00	6 ± 0.00	6 ± 0.00	6 ± 0.00	6 ± 0.00	6 ± 0.00	25 ± 0.50
*Enterococcus faecium* 3934825 HLAR, VRE	6 ± 0.00	6 ± 0.00	6 ± 0.00	16 ± 0.00	6 ± 0.00	6 ± 0.00	6 ± 0.00	6 ± 0.00	6 ± 0.00	25 ± 0.50
*Enterococcus faecium* 773081 HLAR, VRE	6 ± 0.00	6 ± 0.00	6 ± 0.00	17 ± 0.00	6 ± 0.00	6 ± 0.00	6 ± 0.00	6 ± 0.00	6 ± 0.00	25 ± 0.00
*Enterococcus faecium* 895612 HLAR, VRE	6 ± 0.00	6 ± 0.00	6 ± 0.00	15 ± 0.00	35 ± 0.50	20 ± 0.00	6 ± 0.00	6 ± 0.00	20 ± 0.00	26 ± 0.00
*Enterococcus faecium* 508171 HLAR, VRE	6 ± 0.00	6 ± 0.00	6 ± 0.00	15 ± 0.00	40 ± 0.50	6 ± 0.00	6 ± 0.00	6 ± 0.00	6 ± 0.00	24 ± 0.00
*Enterococcus faecium* 830981 HLAR, VRE	6 ± 0.00	6 ± 0.00	6 ± 0.00	6 ± 0.00	6 ± 0.00	6 ± 0.00	6 ± 0.00	6 ± 0.00	6 ± 0.00	25 ± 0.50
*Enterococcus faecium* 264281 HLAR, VRE	6 ± 0.00	6 ± 0.00	6 ± 0.00	6 ± 0.00	6 ± 0.00	6 ± 0.00	6 ± 0.00	6 ± 0.00	6 ± 0.00	25 ± 0.00
*Enterococcus faecium* 967321 HLAR, VRE	6 ± 0.00	6 ± 0.00	6 ± 0.00	15 ± 0.00	35 ± 0.50	6 ± 0.00	6 ± 0.00	6 ± 0.00	6 ± 0.00	25 ± 0.00
*Enterococcus faecium* 966351 HLAR, VRE	6 ± 0.00	6 ± 0.00	6 ± 0.00	16 ± 0.00	6 ± 0.00	6 ± 0.00	6 ± 0.00	6 ± 0.00	6 ± 0.00	26 ± 0.00
*Enterococcus faecium* 576181 HLAR, VRE	6 ±0.00	6 ± 0.00	6 ± 0.00	18 ± 0.00	40 ± 0.50	18 ± 0.00	6 ± 0.00	6 ± 0.00	6 ± 0.00	25 ± 0.50
*Enterococcus faecium* 885041 HLAR, VRE	6 ±0.00	6 ± 0.00	6 ± 0.00	6 ± 0.00	6 ± 0.00	6 ± 0.00	6 ± 0.00	6 ± 0.00	6 ± 0.00	24 ± 0.00

The results are presented as mean values ± standard deviation (±SD) from three independent experiments. Error bars represent standard deviation. HLAR (high-level aminoglycoside resistance), VRE (vancomycin-resistant *Enterococcus*).

**Table 2:** MIC values of cinnamic acid derivatives against *Enterococcus* spp. VRE.

Compd	MIC [µg/mL]							
	*Enterococcus* sp. 773081	*Enterococcus* sp. 508171	*Enterococcus* sp. 830981	*Enterococcus* sp. 264281	*Enterococcus* sp. 967321	*Enterococcus* sp. 966351	*Enterococcus* sp. 576181	*Enterococcus* sp. 885041
**16a**	4 ± 0.21	4 ± 0.18	4 ± 0.20	4 ± 0.15	4 ± 0.22	4 ± 0.30	4 ± 0.30	4 ± 0.33
**16b**	8 ± 0.30	8 ± 0.50	16 ± 0.48	8 ± 0.18	8 ± 0.25	4 ± 0.30	16 ± 0.38	8 ± 0.42
**16c**	4 ± 0.22	4 ± 0.38	4 ± 0.41	4 ± 0.24	4 ± 0.38	4 ± 0.31	4 ± 0.35	4 ± 0.30
**16d**	2 ± 0.10	2 ± 0.10	2 ± 0.10	2 ± 0.05	2 ± 0.15	2 ± 0.10	2 ± 0.22	2 ± 0.10
**16e**	16 ± 0.15	8 ± 0.50	16 ± 0.50	16 ± 0.50	16 ± 0.50	31.25 ± 0.50	8 ± 0.41	8 ± 0.50
**16f**	8 ± 0.45	4 ± 0.25	4 ± 0.18	8 ± 0.50	4 ± 0.25	4 ± 0.28	4 ± 0.28	4 ± 0.35
**17a**	8 ± 0.45	16 ± 0.50	16 ± 0.52	8 ± 0.50	16 ± 0.56	16 ± 0.50	16 ± 0.50	4 ± 0.20
**17b**	>125	>125	>125	>125	>125	>125	>125	>125
**17c**	2 ± 0.18	4 ± 0.25	4 ± 0.25	4 ± 0.19	2 ± 0.05	4 ± 0.25	2 ± 0.25	4 ± 0.25
**17d**	>125	>125	>125	>125	>125	>125	>125	>125
co-trimoxazole	>2000:400	2000:400 ± 0.50	>2000:400	>2000: 400	>2000:400	1250:250 ± 0.50	>2000:400	>2000:400

The results are presented as mean values ± standard deviation (±SD).

**Table 3:** The results of the bacteriostatic effect of the tested compounds in different concentrations on *Enterococcus faecalis* 12245. Bacterial density is given in CFU/mL of sample.

Concentration (μg/mL)	0.5	1	2	4	8	16
**16a**	[1.08 ± 0.65] × 10^8^	[9.00 ± 0.35] × 10^7^	[7.00 ± 0.14] × 10^7^	[6.50 ± 0.10] × 10^7^	[5.00 ± 0.21] × 10^7^	[5.10 ± 0.35] × 10^7^
**16c**	[2.00 ± 0.35] × 10^8^	[9.30 ±0.14] × 10^7^	[8.30 ± 0.14] × 10^7^	[5.90 ± 0.21] × 10^7^	[5.90 ± 0.24] × 10^7^	[4.10 ± 0.35] × 10^7^
**16d**	[1.80 ± 0.10] × 10^8^	[8.20 ± 0.10] × 10^7^	[8.16 ± 0.13] × 10^7^	[4.20 ± 0.14] × 10^7^	[3.90 ± 0.42] × 10^7^	[3.50 ± 0.77] × 10^7^
**16f**	[1.59 ± 0.17] × 10^8^	[1.27 ± 0.30] × 10^8^	[5.70 ± 0.14] × 10^7^	[4.40 ± 0.21] × 10^7^	[2.60 ± 0.20] × 10^7^	[2.00 ± 0.45] × 107

t_0_ = (2.00 ± 0.69) × 10^8^; t_24_ = (1.44 ± 0.47) × 10^8^. The results are presented as mean values ± standard deviation (±SD) from three independent experiments. *p* < 0.05 was considered as statistically significant.

**Table 4:** The results of the bacteriostatic effect of the tested compounds in different concentrations on *Enterococcus faecium* 264281. Bacterial density is given in CFU/mL of sample.

Concentration (µg/mL)	0.5	1	2	4	8	16
**16a**	[1.80 ± 0.28] × 10^8^	[1.30 ± 0.85] × 10^7^	[1.30 ± 0.41] × 10^7^	[1.30 ± 0.70] × 10^7^	[1.20 ± 0.70]× 10^7^	[3.07 ± 0.64] × 10^7^
**16c**	[1.90 ± 0.23] × 10^8^	[3.00 ± 0.35] × 10^7^	[3.00 ± 0.71] × 10^7^	[3.10 ± 084] × 10^7^	[8.00 ± 0.21] × 10^6^	[7.00 ± 0.71] × 10^6^
**16d**	[1.50 ± 0.56] × 10^8^	[1.10 ± 0.60] × 10^7^	[7.00 ± 0.77] × 10^6^	[6.00 ± 0.71] × 10^6^	[5.40 ± 0.42] × 10^6^	[1.20 ± 0.55] × 10^6^
**17c**	[2.27 ± 0.16] × 10^8^	[1.59 ± 0.43] × 10^8^	[1.15 ± 0.58] × 10^8^	[4.40 ± 0.41] × 10^7^	[2.60 ± 0.35] × 10^7^	[2.00 ± 0.62] × 10^7^

t_0_ = (7.30 ± 0.67) × 10^8^; t_24_ = (1.30 ± 0.48) × 10^8^. The results are presented as mean values ± standard deviation (±SD) from three independent experiments. *p* < 0.05 was considered as statistically significant.

**Table 5:** Interactions of cinnamic acid derivatives with antibiotics on resistant strains of *Enterococcus* spp. HLAR.

Strains	Antibiotic	MIC Antibiotic (mg/mL)		Cinnamic Acid Derivative	MIC Cinnamic Acid Derivative (mg/mL)		FICI	Outcome
		Alone	Comb.		Alone	Comb.		
*Enterococcus faecalis* 12214	ampicillin	10	0.001	16a	0.004	0.0005	0.126	synergy
			0.001	16c	0.004	0.0005	0.126	synergy
			0.002	16d	0.001	0.001	1	additivity
			0.001	16f	0.004	0.0005	0.126	synergy
	gentamycin	2	0.001	16a	0.004	0.002	0.5	synergy
			0.001	16c	0.004	0.0005	0.125	synergy
			0.001	16d	0.0005	0.0005	1	additivity
			0.001	16f	0.002	0.0005	0.25	synergy
	streptomycin	10	0.001	16a	0.004	0.002	0.25	synergy
			0.001	16c	0.004	0.0005	0.126	synergy
			0.001	16d	0.002	0.001	0.5	synergy
			0.001	16f	0.002	0.0005	0.25	synergy
*Enterococcus faecalis* 12338	ampicillin	0.8	0.001	16a	0.004	0.0005	0.126	synergy
			0.001	16c	0.004	0.0005	0.126	synergy
			0.001	16d	0.002	0.0005	0.375	synergy
			0.001	16f	0.004	0.0005	0.126	synergy
	gentamycin	2	0.001	16a	0.008	0.002	0.25	synergy
			0.001	16c	0.008	0.0005	0.062	synergy
			0.001	16d	0.001	0.001	1	additivity
			0.001	16f	0.004	0.001	0.25	synergy
	streptomycin	10	0.001	16a	0.004	0.001	0.25	synergy
			0.001	16c	0.002	0.001	0.5	synergy
			0.001	16d	0.001	0.0005	0.5	synergy
			0.002	16f	0.002	0.0005	0.25	synergy

Comb.—combination: compound + antibiotic or antibiotic + compound.

**Table 6:** Interactions of cinnamic acid derivatives with antibiotics on resistant strains of *Enterococcus* spp. VRE.

Strains	Antibiotic	MIC Antibiotic (mg/mL)		Cinnamic Acid Derivative	MIC Cinnamic Acid Derivative (mg/mL)		FICI	Outcome
		Alone	Comb.		Alone	Comb.		
*E. faecium* 966351 VRE	Ampicillin	0.8	0.016	16a	0.008	0.0005	0.082	synergy
			0.008	16c	0.016	0.0005	0.041	synergy
			0.002	16d	0.002	0.0005	0.252	synergy
			0.016	17c	0.016	0.0005	0.082	synergy
	Gentamycin	2	0.001	16a	0.004	0.001	0.25	synergy
			0.001	16c	0.002	0.001	0.5	synergy
			0.001	16d	0.001	0.001	1	additivity
			0.032	17c	0.008	0.008	1.016	additivity
	Streptomycin	10	0.001	16a	0.004	0.002	0.5	synergy
			0.032	16c	0.008	0.008	1.003	additivity
			0.001	16d	0.001	0.0005	0.5	synergy
			0.032	17c	0.008	0.008	1.003	additivity
	Vancomycin	0.008	0.001	16a	0.004	0.0005	0.25	synergy
			0.001	16c	0.002	0.001	0.625	partial synergism
			0.001	16d	0.001	0.001	1.125	additivity
			0.001	17c	0.008	0.0005	0.188	synergy
*E. faecium* 830981 VRE	Ampicillin	0.5	0.016	16a	0.008	0.0005	0.082	synergy
			0.008	16c	0.016	0.0005	0.041	synergy
			0.002	16d	0.002	0.0005	0.252	synergy
			0.016	17c	0.016	0.0005	0.082	synergy
	Gentamycin	2	0.016	16a	0.002	0.0005	0.33	synergy
			0.016	16c	0.001	0.0005	0.58	partial synergism
			0.008	16d	0.001	0.001	1	additivity
			0.001	17c	0.008	0.0005	0.063	synergy
	Streptomycin	5	0.001	16a	0.002	0.002	1	additivity
			0.001	16c	0.002	0.001	0.5	synergy
			0.001	16d	0.001	0.0005	0.5	synergy
			0.002	17c	0.008	0.001	0.125	synergy
	Vancomycin	1	0.002	16a	0.004	0.0005	0.126	synergy
			0.002	16c	0.004	0.0005	0.126	synergy
			0.001	16d	0.001	0.0005	0.5	synergy
			0.001	17c	0.016	0.004	0.251	synergy

Comb.—combination: compound + antibiotic or antibiotic + compound.

**Table 7:** (**a**) List of full names of strains tested in our previous study [18] and in the present study. (**b**) List of full names of strains tested in this publication only.

(**a**)	
**List of Strains Listed in the Publication** [18]	**Full Strain Name and Specific Resistance Mechanism**
*Enterococcus hirae* ATCC 10541	
*Enterococcus faecalis* ATCC 51299	*Enterococcus faecalis* ATCC 51299 VRE
*Enterococcus* sp. 12835	*Enterococcus faecium* 12835 HLAR
*Enterococcus* sp. 12848	*Enterococcus faecium* 12848 HLAR
*Enterococcus* sp. 12214	*Enterococcus faecalis* 12214 HLAR
*Enterococcus* sp. 12245	*Enterococcus faecalis* 12245 HLAR
*Enterococcus* sp.12961	*Enterococccus faecium* 12961HLAR
*Enterococcus* sp. 12338	*Enterococcus faecalis* 12338 HLAR
*Enterococcus* sp. 16247	*Enterococcus faecium* 16247 HLAR
*Enterococcus faecalis* 3937152	*Enterococcus faecalis* 3937152 HLAR, VRE
*Enterococcus faecium* 3934825	*Enterococcus faecium* 3934825 HLAR, VRE
(**b**)	
**List of Strains Listed Only in This Publication**	**Full Strain Name, and Specific Resistance Mechanism**
*Enterococcus* sp. 773081	*Enterococcus faecium* 773081 HLAR, VRE
*Enterococcus* sp. 508171	*Enterococcus faecium* 508171 HLAR, VRE
*Enterococcus* sp. 830981	*Enterococcus faecium* 830981 HLAR, VRE
*Enterococcus* sp. 264281	*Enterococcus faecium* 264281 HLAR, VRE
*Enterococcus* sp. 967321	*Enterococcus faecium* 967321 HLAR, VRE
*Enterococcus* sp. 966351	*Enterococcus faecium* 966351 HLAR, VRE
*Enterococcus* sp. 576181	*Enterococcus faecium* 576181 HLAR, VRE
*Enterococcus* sp. 885041	*Enterococcus faecium* 885041 HLAR, VRE

HLAR (high-level aminoglycoside resistance), VRE (vancomycin-resistant *Enterococcus*).

## Data Availability

Data are contained within the article.
